# Cardiac hypertrophy in a dish: a human stem cell based model

**DOI:** 10.1242/bio.052381

**Published:** 2020-09-21

**Authors:** Markus Johansson, Benjamin Ulfenborg, Christian X. Andersson, Sepideh Heydarkhan-Hagvall, Anders Jeppsson, Peter Sartipy, Jane Synnergren

**Affiliations:** 1 Systems Biology Research Center, School of Bioscience, Department for Biology and Bioinformatics, University of Skövde, SE-541 28 Skövde, Sweden; 2 Department of Molecular and Clinical Medicine, Institute of Medicine, The Sahlgrenska Academy at University of Gothenburg, 405 30 Gothenburg, Sweden; 3 Takara Bio Europe AB, 413 46 Gothenburg, Sweden; 4 Bioscience, Research and Early Development, Cardiovascular, Renal and Metabolism (CVRM), BioPharmaceuticals, R&D AstraZeneca, 431 50 Gothenburg, Sweden; 5 Department of Cardiothoracic Surgery, Sahlgrenska University Hospital, 413 45 Gothenburg, Sweden; 6 Late-stage Development, Cardiovascular, Renal and Metabolism (CVRM), BioPharmaceuticals R&D, AstraZeneca, 431 50 Gothenburg, Sweden

**Keywords:** Cardiac hypertrophy, Cardiomyocytes, Disease model, Endothelin-1, Stem cells

## Abstract

Cardiac hypertrophy is an important and independent risk factor for the development of heart failure. To better understand the mechanisms and regulatory pathways involved in cardiac hypertrophy, there is a need for improved *in vitro* models. In this study, we investigated how hypertrophic stimulation affected human induced pluripotent stem cell (iPSC)-derived cardiomyocytes (CMs). The cells were stimulated with endothelin-1 (ET-1) for 8, 24, 48, 72, or 96 h. Parameters including cell size, ANP-, proBNP-, and lactate concentration were analyzed. Moreover, transcriptional profiling using RNA-sequencing was performed to identify differentially expressed genes following ET-1 stimulation. The results show that the CMs increase in size by approximately 13% when exposed to ET-1 in parallel to increases in ANP and proBNP protein and mRNA levels. Furthermore, the lactate concentration in the media was increased indicating that the CMs consume more glucose, a hallmark of cardiac hypertrophy. Using RNA-seq, a hypertrophic gene expression pattern was also observed in the stimulated CMs. Taken together, these results show that hiPSC-derived CMs stimulated with ET-1 display a hypertrophic response. The results from this study also provide new molecular insights about the underlying mechanisms of cardiac hypertrophy and may help accelerate the development of new drugs against this condition.

## Abstract (precis)

**Summary:** This research describes a functional *in vitro* model of cardiac hypertrophy, and how the time of stimulation with endothelin-1 affects the cells.

## INTRODUCTION

Cardiac hypertrophy is characterized by an enlargement of the heart due to an increase in size of the cardiomyocytes (CMs) (Frey et al., 2004). There are two major types of cardiac hypertrophy; physiological and pathological. Physiological hypertrophy is naturally occurring during post-natal growth, pregnancy and exercise (Pluim et al., 2000; Eghbali et al., 2005; Janz et al., 2000). It is reversible and does not progress to clinically overt heart failure. Pathological hypertrophy, on the other hand, may progress to heart failure if the stimuli persists for an extended time and is a maladaptive decompensatory condition where the heart increases in size due to alteration in several signaling pathways (Pham et al., 2000; Rockman et al., 2002; Bueno et al., 2000). These alterations result in pathological hypertrophy including adverse gene expression profile, increase in ANP and BNP protein levels, and increased lactate production due to a higher glucose consumption (Frey and Olson, 2003; Almeida et al., 2003; Kolwicz and Tian, 2011). This type of cardiac hypertrophy is induced by conditions such as chronic hypertension, aortic stenosis, myocardial infarction, or gene mutations. Until recently, only animal-based models have been available to study cardiac hypertrophy in a pre-clinical setting. These models are in many ways useful, but there are significant differences between human- and animal-cardiovascular systems, including stress response and ion channel expression, which makes translation to the human situation challenging (Milani-Nejad and Janssen, 2014). Novel technologies, based on human stem cells, could offer clinically relevant *in vitro*-based alternatives.

Human pluripotent stem cells (hPSCs) have a unique capability to self-renew and differentiate into all cell types in the body (Thomson et al., 1998). These features make them useful for various *in vitro* applications, such as toxicity testing and disease modeling. In particular, hPSC-derived CMs have proven to be useful in many *in vitro* assays (Holmgren et al., 2014; Lundin et al., 2018; Geraets et al., 2018). Human cell-based models are anticipated to provide alternatives to the use of animal models for studies of cardiac hypertrophy mechanisms. Besides providing systems that are scalable and may improve the translation of the results to the clinical situation, the availability of human cell-based models can also help to reduce the need for animal experiments.

Cardiac hypertrophy can be induced by different methods *in vitro*, where the most commonly used are neurohormonal stimulation and physical stretching. It is also possible to use hPSC derived from patients carrying genetic mutations that cause cardiac hypertrophy and differentiate these cells into cardiomyocytes (Zhao et al., 2019). Another approach is to genetically modify hPSC so that the cells when differentiated into cardiomyocytes gain a phenotype that resembles cardiac hypertrophy (Xie et al., 2019).

The neurohormonal approach uses substances that bind to specific receptors and activate signaling pathways that initiate changes in a series of compensatory mechanisms, involving heart rate, heart contractility, and salt and water retention (Wu et al., 2006; Kim and Iwao, 2000). All for trying to maintain the cardiovascular homeostasis. Commonly used substances for cardiac hypertrophy induction are ET-1 and phenylephrine (Foldes et al., 2011; Tanaka et al., 2014; Aggarwal et al., 2014; Deisl et al., 2019; Carlson et al., 2013). Both of these are vasoconstrictors; ET-1 being the most potent one (Davenport et al., 2016).

The stretch model uses mechanical force to induce hypertrophy. Several extracellular matrix sensing receptors, e.g. integrins and cytoskeletal filaments activate signaling pathways, which trigger a hypertrophic response (Ovchinnikova et al., 2018). It is well established that this type of stimuli results in a hypertrophic response with increased cell size and upregulation of hypertrophic markers (Foldes et al., 2011; Ovchinnikova et al., 2018).

Genetically modified hPSC-CMs show promising results. With this method, a specific gene, or set of genes, that are known to cause hypertrophy are identified and then CRISPR/CAS9 could be employed to modify the cells. Recently, mutations in ADAM17 have been linked to the development of Tetralogy of Fallot, a condition with ventricular hypertrophy as an outcome. hPSC-CMs with these mutations in ADAM17 have been shown to develop hypertrophy (Xie et al., 2019). It should also be noted that there are also more complex *in vitro* systems for cardiac hypertrophy. For example, a platform for generation of chamber specific tissues and disease modeling was recently shown to serve as an *in vitro* model for cardiac hypertrophy. In this model, patient specific hiPSC-CMs were exposed to chronical electrical conditioning for an extended time period (months) which led to the development of a hypertrophic phenotype (Zhao et al., 2019).

All of the mentioned approaches have been shown to render a hypertrophic state in hPSC-derived CMs. However, the degree of response varies between studies. The different experimental settings used, including starting material, culture medium and the induction method, may help to explain these differences and there is a need for standardization between laboratories (Aggarwal et al., 2014; Tanaka et al., 2014; Foldes et al., 2011; Ovchinnikova et al., 2018). Notably, serum used in the culture medium can induce hypertrophy in cultured CMs, which needs to be considered when designing hypertrophy experiments and ideally, investigators should avoid using serum supplemented culture medium or only use serum at a very low concentration (Dambrot et al., 2014).

Currently, the reports on neurohormonal *in vitro* models for cardiac hypertrophy show promising results with increased cell size and a hypertrophic gene expression pattern when using ET-1, phenylephrine or angiotensin II (Aggarwal et al., 2014; Foldes et al., 2011; Ovchinnikova et al., 2018; Deisl et al., 2019; Carlson et al., 2013). However, the response varies depending on substance used. For example, angiotensin II does not seem to result in the same cell size increase as caused by phenylephrine and ET-1 (Foldes et al., 2011). Neurohormonal hypertrophy induction has been accomplished in hPSC-CMs derived from many different cell lines and the effect appears to be cell line independent (Carlson et al., 2013; Aggarwal et al., 2014; Foldes et al., 2011; Deisl et al., 2019). Comparing *in vitro* changes of gene expression induced by ET-1 to the expression changes in left ventricular hypertrophy *in vivo* provides strong evidence that this type of model is reflecting the *in vivo* condition in humans (Aggarwal et al., 2014). However, they thus far lack information on how the hypertrophy is regulated over time. The stem cell-based models described to date typically use single time point stimulations and responses are usually analyzed within 24 h (Foldes et al., 2011; Aggarwal et al., 2014). Such models are suitable for assessing the acute responses but they do not provide information on how the hypertrophy response develops over time. Another critical component of the model is the cell source, as the CMs need to be of high quality and purity while possessing key cardiac functionalities. Extended culturing time has been shown some improvement in the maturation of the hiPSC-CMs and is therefore an important parameter to consider when developing an *in vitro* model based on such cells (Dias et al., 2018).

In this study, we have investigated the hypertrophic response over time in hiPSC-derived CMs. The CMs were stimulated with ET-1 during various time points and assessed the acute and long-term hypertrophic response were subsequently analyzed.

## RESULTS

### Cardiomyocyte homogeneity

The CMs obtained from Takara Bio Europe have been differentiated from the ChiPS22 cell line for 19 and 32 days respectively. The differentiated cultures of CMs were homogenous and showed a high proportion of cardiac troponin T (cTnT) positive cells and displayed spontaneous and synchronous beating throughout the experiments.

**Fig. 1.**
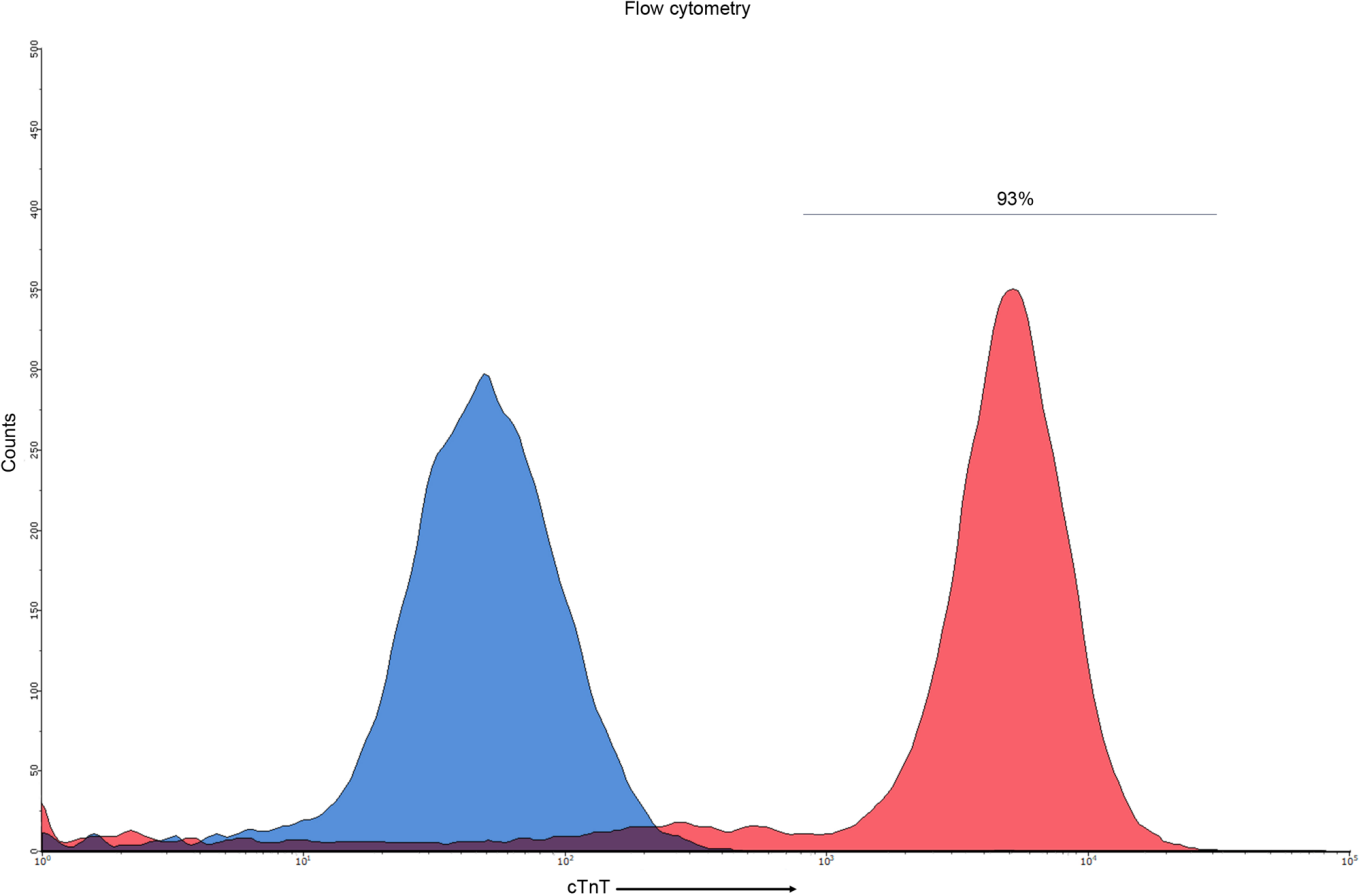
**Histogram plot of the FC analysis performed on the CMs used in the study.** The x-axis shows the number of counts and the y-axis shows the fluorescence intensity. Blue peak represents control and red peak represents the sample. The bar signifies the interval of cTnT+ cells.

The homogeneity of the CM cultures was analyzed with flow cytometry (FC) before cryopreservation at day 19 and 32 following start of differentiation. The results show that, prior to cryo-preservation at day 19, 97% of the cells were cTnT positive (data not shown) and the population of cells at day 32 contained 93% (range 91–96%) cTnT positive cells (Fig. 1). The percentage of cTnT+ cells of the control cells, undifferentiated cells of ChiPS22, was <0.5%. Hereinafter the day 19 and 32 CMs will be named day 25 and 38, respectively, because of the 6 day culture period that was used after thawing in all the experiments.

**Fig. 2.**
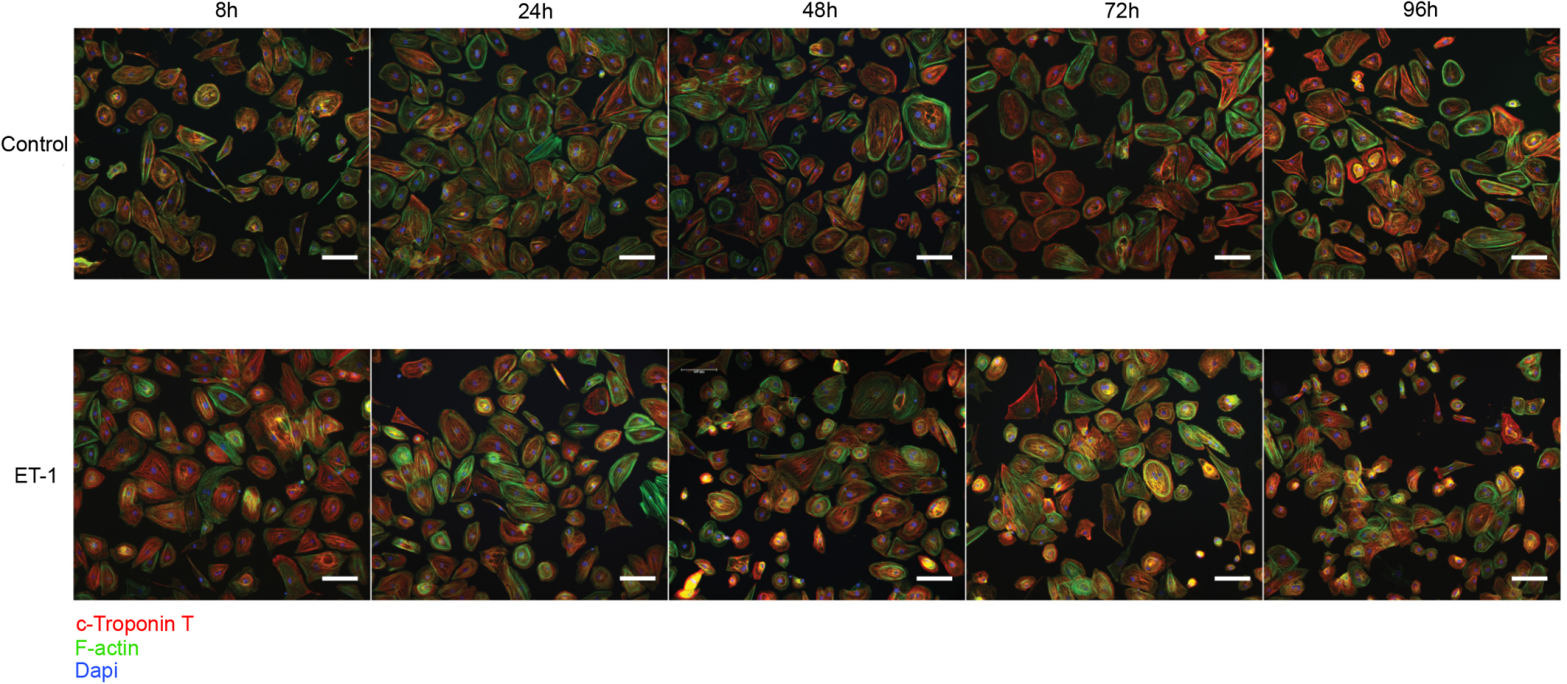
**ICC images of control and ET-1 stimulated CMs.** Cardiomyocytes stained for c-troponin T (red), F-actin (green) and Dapi (blue). The first row of images shows control CMs and the second row ET-1 stimulated CMs, both groups at 8, 24, 48, 72 and 96 h. Images were captured at 10× and the scale bars represents 100 µm.

In order to monitor the cells after thawing and ET-1 stimulation, we also performed immunocytochemistry (ICC) and stained the day 38 CMs for cTnT (Red) and F-actin (Green) (Fig. 2). Images were captured at 8, 24, 48, 72, and 96 h after ET-1 stimulation. Images of control cells (incubated without ET-1) were also captured. The ICC analysis verified the FC results and demonstrates a high proportion of CMs in the cultures. Based on image observation, no change with regard to homogeneity and CM composition could be detected in control or ET-1 stimulated CMs over the 96 h time period. In summary, the CM population remained stable over time and no difference in expression of cTnT could be observed over the course of the experiments.

### Gene expression

**Fig. 3.**
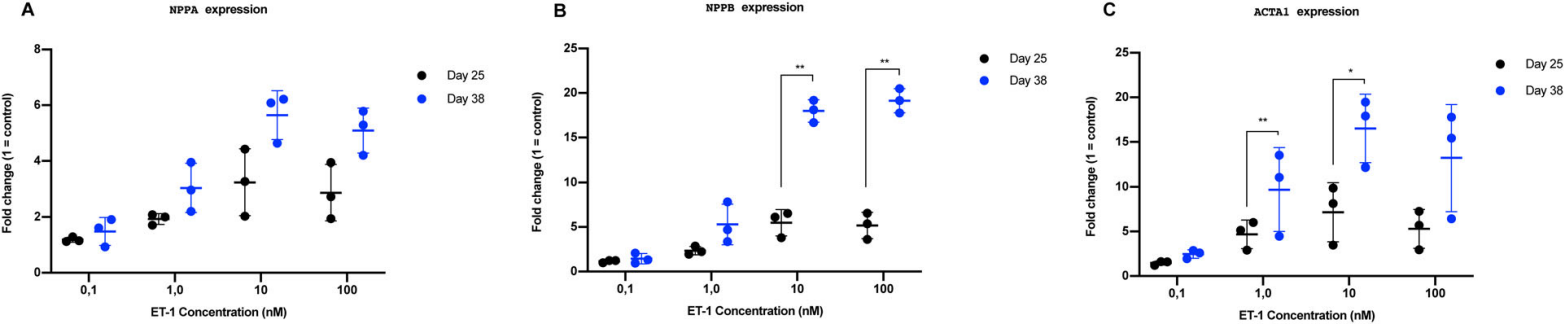
**RT-qPCR results from dose response experiment using CMs cultured to day 25 and day 38.** The results are presented as relative fold change, normalized against the endogenous control CREBBP and unstimulated control CMs. (A) NPPA expression (B) NPPB expression (C) ACTA1 expression. Individual values are represented as black dots (day 25) and blue dots (day 38). Standard deviation (s.d.) are given as error bars (*n*=3). *P<0.05, **P<0.01.

The response to ET-1 were both dependent on concentration and affected by the time in culture of the CMs. To determine the optimal concentration of ET-1, dose-response experiments were conducted and the gene expression levels of three commonly used hypertrophy markers (*NPPA*, *NPPB* and *ACTA1*) were analyzed (Fig. 3). The experiments were performed on CMs cultured for 25 and 38 days after initiation of differentiation, in order to investigate if the time in culture had any effect on the hypertrophic response. The concentrations 0.1, 1.0, 10 and 100 nM of ET-1 were used.

After ET-1 stimulation for 24 h, *NPPA*, *NPPB* and *ACTA1* levels were all upregulated in a dose-dependent manner. Maximal response was observed at 10 nM ET-1. Notably, the response was more pronounced in CMs cultured for 38 days compared to cells at day 25 (Fig. 3A–C). The gene expressions of *NPPB* and *ACTA1* were 9-fold and 12-fold increased, respectively, at 10 nM at day 38 compared to day 25 (*P*<0.05) (Fig. 3B,C). The expression of *NPPA* was 2-fold higher at 10 nM at day 38 compared to day 25; however, it was not statistically significant (Fig. 3A). Taken together, these data demonstrate that CMs cultured for 38 days produce a more robust hypertrophic response, as measured by gene expressions of key markers, compared to CMs at day 25. Based on these results, day 38 CMs and 10 nM of ET-1 was used subsequently in all following experiments in the study.

### Protein analysis

ET-1 stimulated CMs show an increased secretion of the natriuretic proteins ANP and proBNP.

**Fig. 4.**
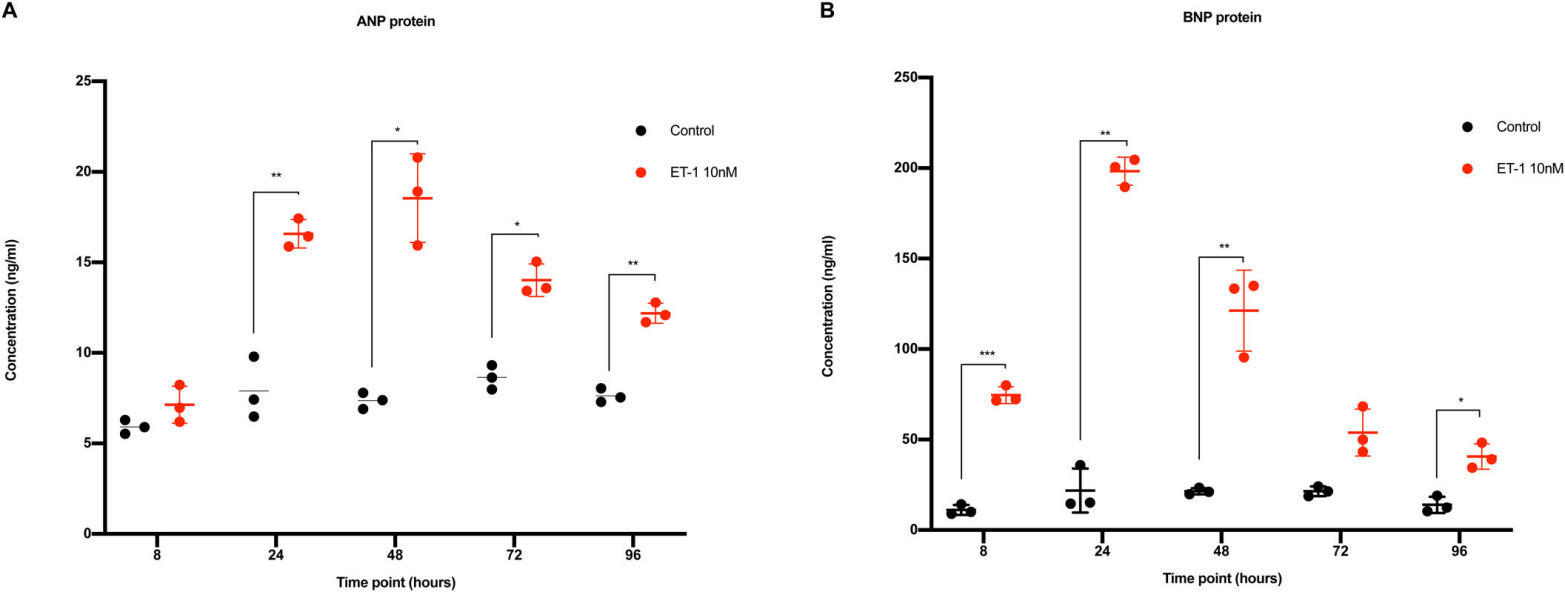
**ANP and proBNP analysis in conditioned media.** Red dots represent CMs stimulated with ET-1 and black dots represent control CMs. The x-axis shows the time points and the y-axis the concentration in ng/ml. (A) Concentration of secreted ANP protein. (B) Concentration of secreted proBNP protein. s.d. is given as error bars (*n*=3). *P<0.05, **P<0.01, ***P<0.001.

Incubation with ET-1 significantly upregulated the expression of ANP between 24 and 96 h. After 8 h of stimulation, there was no difference in ANP between control and ET-1 stimulated CMs. The increase was evident from 24 h and onward. The highest concentration of ANP was observed after 48 h and the concentration was more than 2-fold compared to the controls. After 48 h and onwards, the ANP-concentration decreased slightly with each time point. However, still at 96 h, the concentration of ANP was significantly higher compared to control. The control CMs showed a stable level of ANP protein during the entire experiments (Fig. 4A).

Furthermore, proBNP was significantly upregulated at all the time points studied (8–96 h), except for the 72 h time point, which did not reach statistical significance (*P*=0.056). Already after 8 h the concentration of proBNP was more than 6-fold higher compared to controls. The maximum concentration was observed at 24 h, representing a 9-fold increase compared to control. Repeated stimulation after 24 h did not further increase the proBNP concentration. Instead, a decay at every time point after 24 h was observed. Although decreased, the concentration was still significantly higher compared to control at the last time point studied (96 h) (Fig. 4B). The control CMs showed a stable level of proBNP protein during the entire experiments (Fig. 4A,B).

### Lactate analysis

**Fig. 5.**
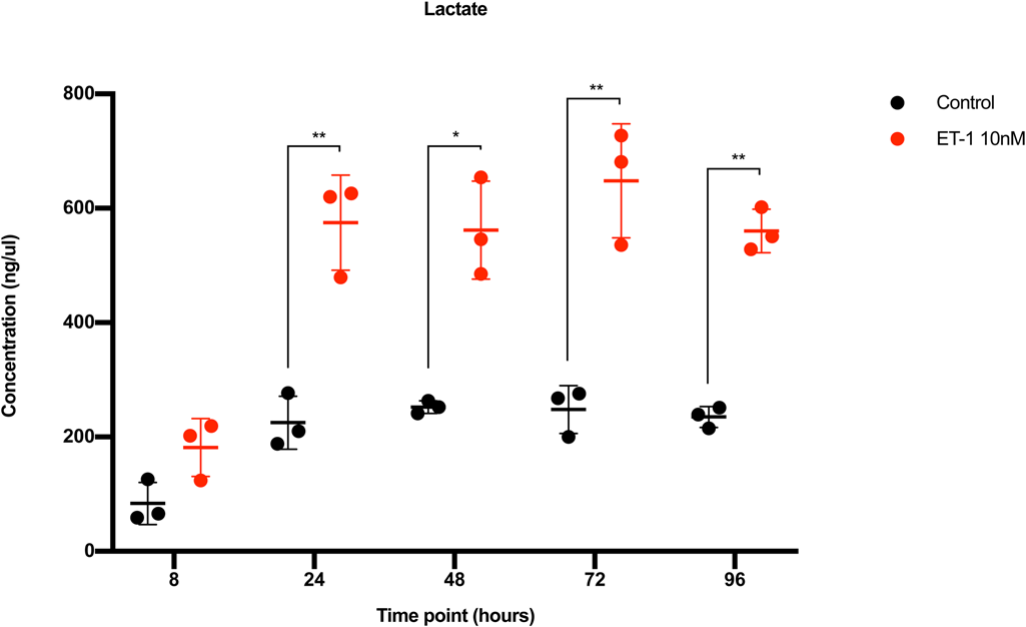
**Lactate concentration analysis in conditioned media.** The x-axis shows the time points and the y-axis the concentration of lactate in ng/µl. Red dots represent CMs stimulated with ET-1 and black dots represent control CMs. s.d. is given as error bars (*n*=3). *P<0.05, **P<0.01.

The lactate concentration in the culture media was significantly increased in the ET-1 stimulated CMs. Lactate concentration is an indirect measurement of the glucose consumption. Increased glucose consumption is an important feature of the hypertrophy response. The lactate concentration in the ET-1 stimulated CMs was over 2.5-fold compared to control cells at the 24, 48, 72 and 96 h time points (Fig. 5). The mean lactate concentration in the media at those time points were 586 ng/µl (479–727 ng/µl) compared to 240 ng/µl (188–247 ng/µl) in the controls (*P*<0.0001). An increase was observed already at the 8 h time point, albeit not statistically significant. The rate of which lactate was produced was similar at all time points, indicating that the increase in glucose consumption is an immediate effect of stimulation with ET-1.

### Cell size

**Fig. 6.**
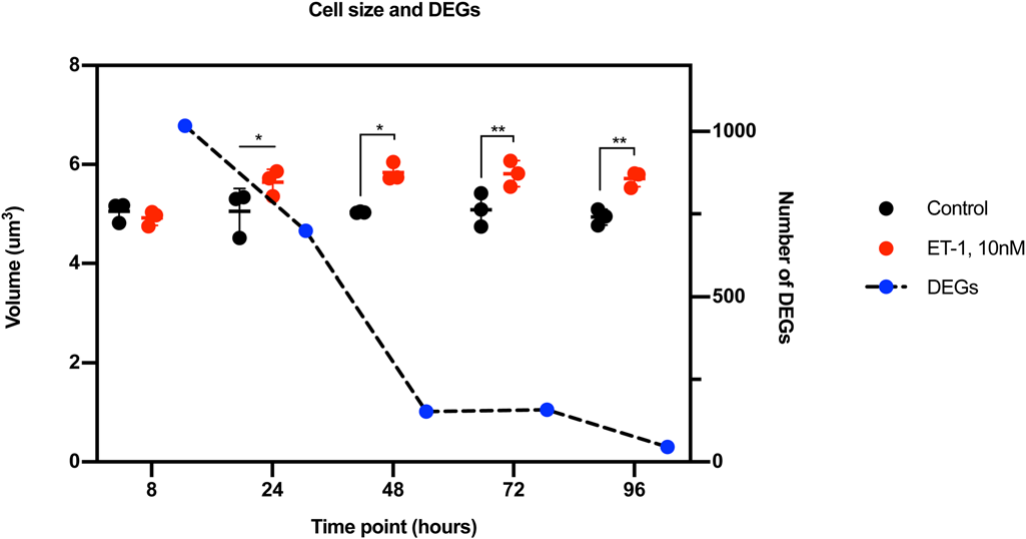
**The graph shows the cell size and the number of DEGs.** The dots represent the cell volume and the squares represent DEGs. Black dots are controls and red dots represent stimulated cells. The blue dots with a black dotted line show the number of DEGs. The left y-axis shows the volume, the right y-axis the number of DEGs, and the x-axis the different time points.

The increase in CM cell size was evident after 24 h of stimulation with ET-1. Cell size was analyzed in living cells at several time points. At 8 h of stimulation with ET-1, there was no significant change in cell size compared to controls. However, at 24 h, the ET-1 stimulated CMs displayed an increased volume compared to 8 h of stimulation [5.64 µm^3^ (5.35–5.86 µm^3^) versus 4.92 µm^3^ (4.75–5.04 µm^3^), *P*<0.01] and was significantly larger than the control CMs, which had a volume of 5.05 µm^3^ (4.51–5.35 µm^3^), *P*=0.04. At 48, 72 and 96 h of stimulation, the ET-1 stimulated CMs had a significantly increased volume by an average of 0.87 µm^3^ (13%) compared to controls (*P*<0.0001) (Fig. 6). No further increase in size was detectable after 48 h. The control CMs remained at approximately the same size at every time point throughout the experiments.

### Transcriptional profiling

Transcriptional profiling of ET-1 stimulated CMs revealed a robust hypertrophic response.

**Fig. 7.**
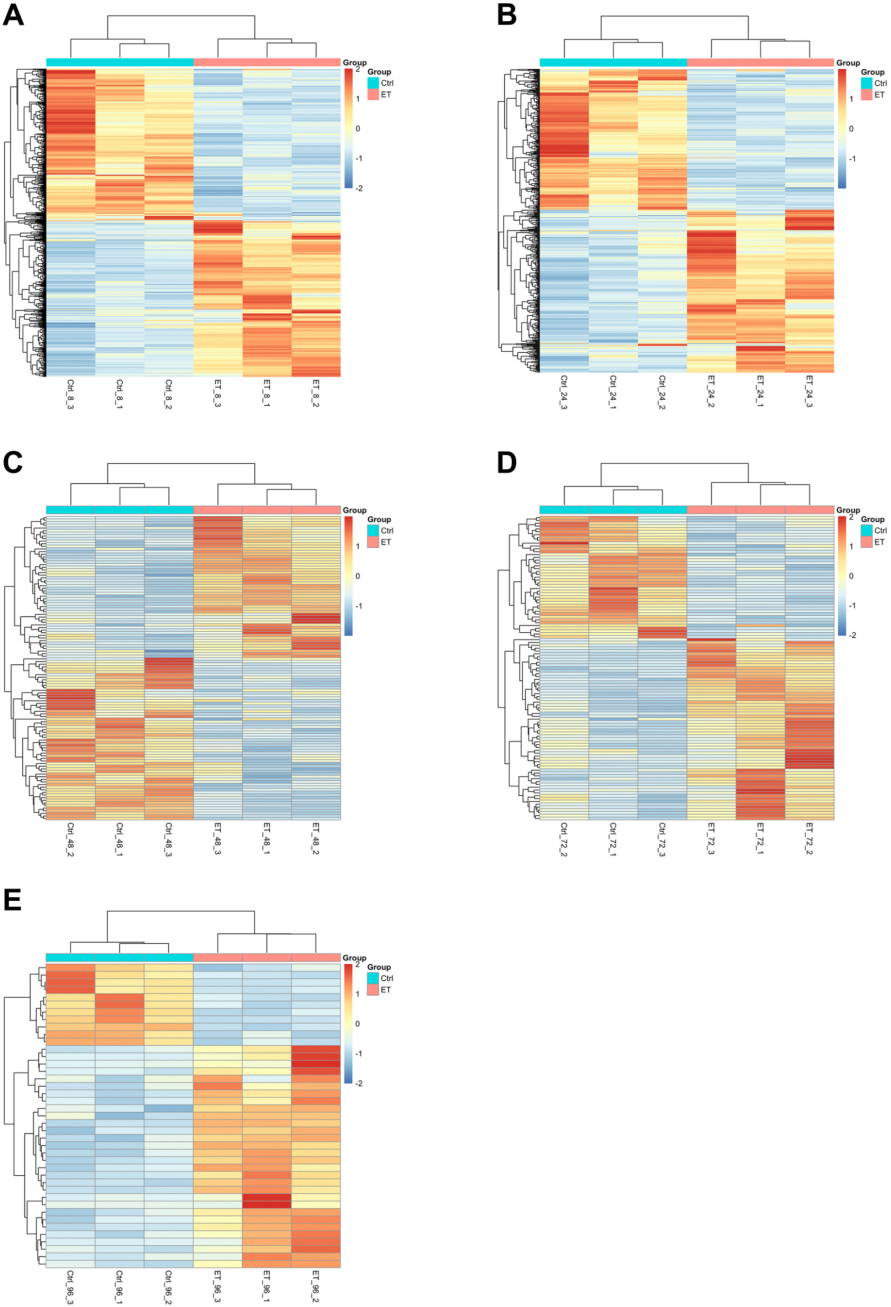
**Heatmaps of all DEGs (rows represent genes and columns represent samples).** The color indicates the scaled expression values with red color representing high expression and blue representing low expression. (A) The total of 1017 DEGs (43% upregulated and 57% downregulated) at 8 h of ET-1 stimulation. (B) The total of 699 DEGs (43% upregulated and 57% downregulated) at 24 h of ET-1 stimulation. (C) The total of 155 DEGs (45% upregulated and 55% downregulated) at 48 h of ET-1 stimulation. (D) The total of 158 DEGs (68% upregulated and 32% downregulated) at 72 h of ET-1 stimulation. (E) The total of 45 DEGs (73% upregulated and 27% downregulated) at 96 h.

Gene expression profiles obtained by RNA-seq analysis at 8, 24, 48, 72, and 96 h of ET-1 stimulation were investigated. This analysis identified 1017, 699, 152, 158, and 45 differentially expressed (2-fold, *P*<0.05) genes at 8, 24, 48, 72, and 96 h, respectively (Fig. 7). Interestingly, the cell size did not correlate with the number of differentially expressed genes (DEGs). The cell size was maintained from 24 to 96 h while the number of DEGs decreased over this time period (Fig. 6).

Hierarchical clustering shows a clear separation of all the ET-1 treated samples and the control samples. At the 8, 24, and 48 h time points, the numbers of upregulated and downregulated genes were in similar range (Fig. 7A–C). However, at the later time points (72 h and 96 h) the majority of DEGs were upregulated by the ET-1 stimulation (Fig. 7D,E).

**Fig. 8.**
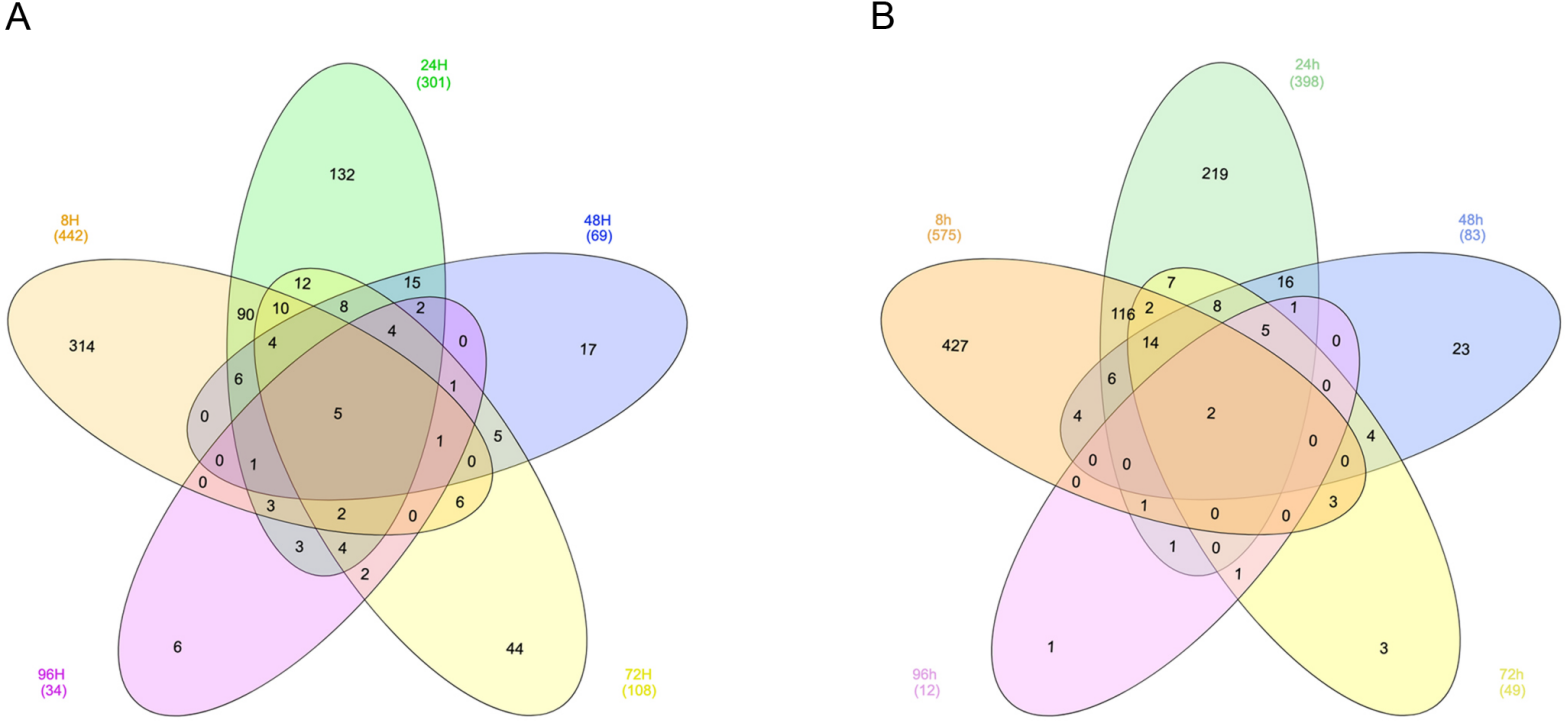
**Venn diagrams showing the number of differentially expressed genes identified after ET-1 stimulation at different time of exposure.** The numbers in parentheses show the total number of upregulated genes (A) and downregulated genes (B) for each exposure time. The numbers indicated in the different areas in the Venn diagrams represent the number of identified genes that overlap across the different exposure times. Five genes are identified as upregulated regardless of exposure time. The corresponding number for the downregulated genes is two. Lists of up- and downregulated genes corresponding to these Venn diagrams are shown in Table S1.

The Venn diagrams (Fig. 8A,B) describe the overlap of significantly upregulated and downregulated genes at the different time points. For the upregulated genes, only five genes were overlapping between all the time points. These genes were *DPYSL4*, *PHACTR3*, *COL12A1*, *NPPA* and *PRF1*. The largest overlap (121 genes) was observed between the 8 h and 24 h time points (Fig. 8A). The same trend was evident for the downregulated genes with only two overlapping genes (DRD1 and GRM1) across all the time points. The highest number of overlapping DEGs that show downregulation (141 genes) was observed between time point 8 h and 24 h (Fig. 8B).

A GO enrichment analysis was performed applying the Cytoscape software and the ClueGO plugin on the list of upregulated genes in each time point, respectively. In addition to enrichment of specific terms related to cardiac hypertrophy, such as muscle structure development, the GO enrichment results show a broad response of various functions from the ET-1 stimulation at the early time points, 8 to 24 h (Fig. S1A,B). At the 24 h time point, there was still a relatively broad response shown among the enriched GO terms but also many GO terms specifically related to cardiac hypertrophy (muscle contraction, extracellular structure organization, myofibril assembly and structural constituent of cytoskeleton) were enriched (Fig. S1B). At the later time points 48, 72 and 96 h, fewer enriched GO terms were observed, but on the other hand, these were highly relevant in terms of the cardiac hypertrophy mechanism (Fig. S1C–E). These results are in line with the data showing a broad response in the cells at the beginning of the ET-1 stimulation and a more specific effect is observed after 48 h of stimulation. This indicates that the CMs to some extent adapt to stimulation of ET-1.

**Fig. 9.**
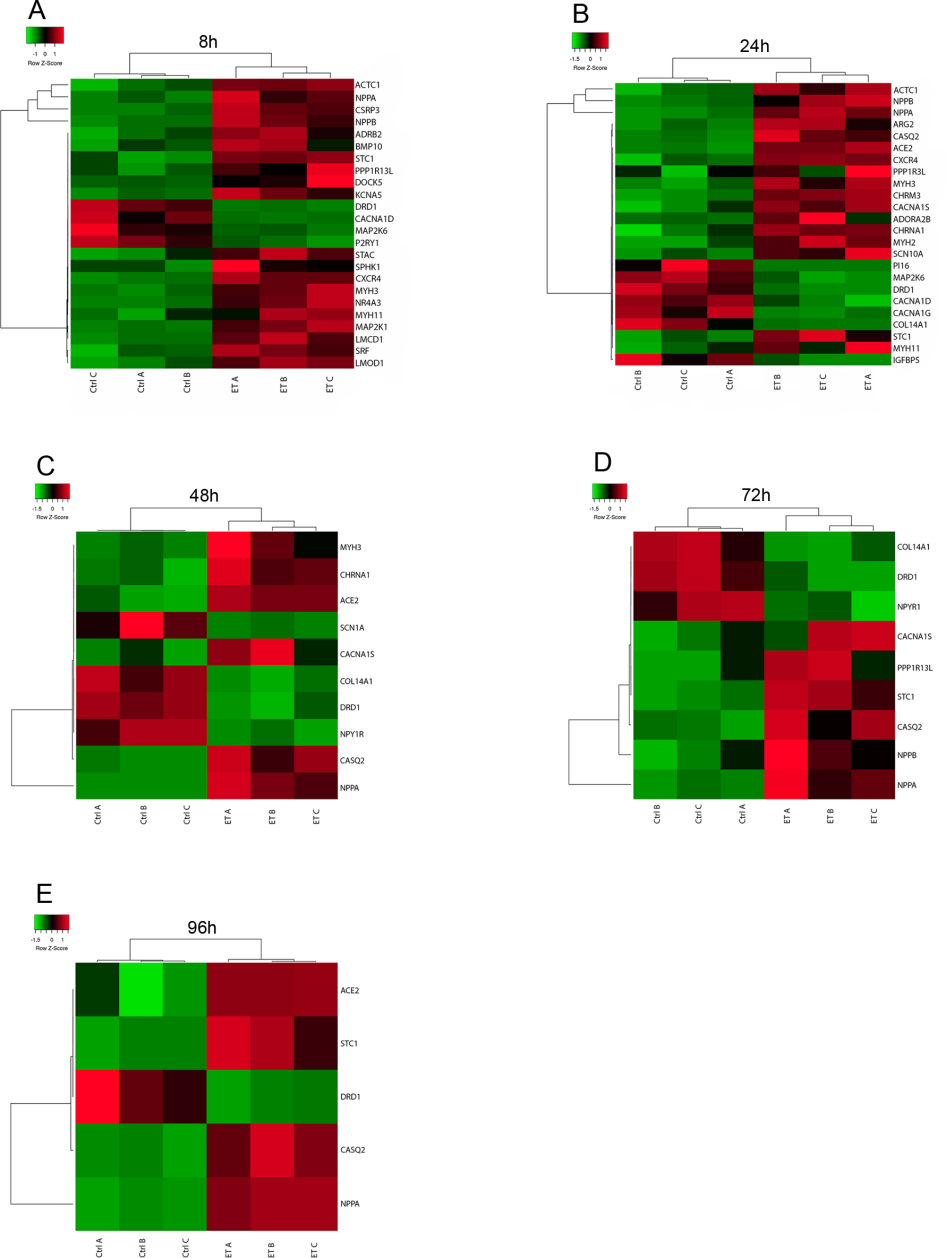
**Heatmaps of DEGs annotated with the GO term Muscle system processes at the different time points and that are identified as significantly differentially expressed.** The scale goes from green to red with the highest z-score as red and the lowest as green. The rows represent the DEGs found among the genes that are annotated with the GO-term Muscle system processes and the columns represent the samples. For all investigated time points, the majority of the identified DEGs show upregulation by ET-1 stimulation. (A) 8 h, (B) 24 h, (C) 48 h, (D) 72 h, (E) 96 h.

To further explore the transcriptional profiles of genes of specific relevance for muscle tissue, 398 genes annotated with the GO term ‘muscle system processes’ (GO level 4) where selected and explored in more detail. The number of genes assigned with this term and that show differential expression in our data was calculated. In total 24, 24, ten, nine, and six genes at the time points 8, 24, 48, 72 and 96 h, respectively, (Fig. 9A–E) were annotated with this GO term and were differentially expressed in the ET-1 stimulated cells. We also explored genes more specifically annotated with ‘cardiac muscle hypertrophy in response to stress’, which are of very high relevance for this disease model such as *NPPA*, *NPPB* and *BMP10*, and these are all differentially expressed in the ET-1 stimulated cells. *NPPA* is differentially expressed at all investigated time points and *NPPB* at 8, 24 and 72 h (Fig. 9A–C). *BMP10* is only differentially expressed at the 8 h time point (Fig. 9A). Cardiac hypertrophy is associated with alterations in calcium handling and hence, it is reassuring that genes encoding for proteins involved in calcium handling and signaling (*CACNA1D*, *CACNA1G*, *CACNA1S* or *CASC2*) also show differential expression at all time points in our model (Fig. 9A–E).

## DISCUSSION

In this study, we have exposed hiPSC-derived CMs to ET-1 and evaluated the response with various methods. The model shows a robust response to ET-1. We identified upregulation at both gene and protein levels, of the natriuretic proteins ANP and proBNP in the stimulated CMs. Physical parameters such as cell size increased during the first 24 h and stayed increased throughout the experiments. The same observation was evident regarding lactate production in the cells. We also used RNA-seq to assess the global gene expression changes in the cells following ET-1 stimulation. Interestingly, a substantial and broad response was observed at the early time points of stimulation. However, the CMs seem to adapt to the ET-1 stimulation over time and the initial broad response transforms into a more focused response characteristic of cardiac hypertrophy.

The starting cell material is of great importance in all cell biology studies and it is known that extended culture of CMs can enhance their functionality (Dias et al., 2018). Therefore, we chose to assess the utility of CMs that had been differentiated and cultured for various time periods (i.e. 25 and 38 days). We performed real-time qPCR analysis and measured the expression of a few key hypertrophy genes (*NPPA*, *NPPB* and *ACTA1*) to elucidate if time in culture affects the hypertrophic response. The results clearly showed a difference in response between the day 25 and day 38 CMs. This is in line with results from other studies where extended culture of the CMs also showed enhanced maturation of the cells (Kamakura et al., 2013; Lundy et al., 2013; Dias et al., 2018). The difference in response related to time in culture can probably be one explanation of why there is a discrepancy among the data reported previously regarding hypertrophic responses in stem cell-derived CMs (Foldes et al., 2014; Lewandowski et al., 2018). Functional data from the CMs used in this study have been described previously. In short, the CMs start beating regularly and synchronized approximately 48 h after thawing/seeding and they display normal action potential profiles. Furthermore, they have been shown to have an expected electrophysiological response to different drugs (Higa et al., 2016; Da Rocha et al., 2017; Bot et al., 2018; Stillitano et al., 2017). Electrophysiological studies performed on iPS-CMs derived from patients with familial hypertrophic cardiomyopathy show that the patient-specific CMs have more irregular action potentials and increased irregular contractility compared to control-CMs (Han et al., 2014). In addition, stem cell-derived CMs used in a stretch model have also been analyzed with regard to their electrophysiological properties, with no observed change in beating force and with slight decrease in beats per minute compared to the controls (unstretched CMs) (Ovchinnikova et al., 2018). In line with these recent studies, we did not observe any differences in beating pattern of the CMs before and after incubation with ET-1.

In this work, we tested four different concentrations of ET-1 (0.1, 1.0, 10 and 100 nM) to determine the optimal concentration for hypertrophy induction. The results show that 10 nM gave optimal response in terms of gene expression of *NPPA*, *NPPB* and *ACTA1*. These results are in line with data from other studies that have used ET-1 to induce hypertrophy (Aggarwal et al., 2014; Zolk et al., 2004; Tanaka et al., 2014). We also tested different durations of ET-1 stimulation between 8 h and 96 h. To our knowledge, this has not previously been reported with hiPSC-derived CMs using the neurohormonal induction method. Using the stretch-induced method, differences have been shown in the response depending on duration of mechanical force. Forty-eight hours seems to be the most suitable duration of stimulation when conducting those types of hypertrophy experiments, resulting in most robust release of the proBNP protein (Ovchinnikova et al., 2018). In our model, the highest amount of proBNP was observed after 24 h. This difference could be due to the induction method and that the neurohormonal approach results in a slightly faster hypertrophic response. However, hiPSC-based models using ET-1 have no comprehensive data available from the very early time of stimulation and most of the studies are conducted between 18 to 48 h of stimulation. Rat models have on the other hand been tested with longer ET-1 stimulation and results from these studies show that the time of stimulation have a great impact on the hypertrophy response (Zolk et al., 2004). In line with this, our results also demonstrate that the time of stimulation have a significant effect of the hypertrophy response also in human cells. Initially, the response is broad and the highest number of differentially expressed genes are identified at the first time point we tested (i.e. 8 h) (Fig. 6). This observation is of importance since it can give more insight into the initiation phase of the hypertrophy response. At this early time point, we could not observe any increase in cell size. The increase in size was initially observed after 24 h, which is also in agreement with results from other studies (Tanaka et al., 2014; Foldes et al., 2011). However, those studies only present data from 24–48 h of stimulation. Possibly, 8 h is not long enough for the CMs to produce and organize the new proteins that are necessary for a cell size increase and that may be the reason why we only observe the effect on a transcriptional level at this time point. Based on this, we can conclude that the time of stimulation is an important factor to consider when developing a hypertrophy disease model. A shorter stimulation is more suitable for investigations of the initial response before any detectable changes in cell size have occurred. Stimulation time points at, or beyond, 24 h appear more appropriate if the aim is to correlate molecular pathways with changes in cell size.

Thus far, there are no reports on how gene expression is regulated over time when hiPSC-derived CMs are stimulated repeatedly from 8 h to 96 h with ET-1. A comparison of all the time points revealed that five genes were upregulated at all time points following stimulation with ET-1: *DPYSL4*, *PHACTR3*, *COL12A1*, *NPPA* and *PRF1*. Of these genes, *NPPA* is a common hypertrophy marker. The *NPPA* gene codes for the ANP protein, which is a natriuretic peptide hormone that regulates cardiac homeostasis through regulation of natriuresis, diuresis and vasodilation. Notably, the NPPA gene is upregulated in ET-1 based *in vitro* models of cardiac hypertrophy (Carlson et al., 2013; Tanaka et al., 2014; Aggarwal et al., 2014; Deisl et al., 2019). The other four genes are not typical cardiac hypertrophy genes. However, *DPYSL4*, *PHACTR3* and *COL12A1* are all likely involved in remodeling of the cytoskeleton (Azevedo et al., 2016). More research is needed to elucidate the cellular consequences in the cardiomyocytes when these genes are upregulated. *DRD1* and *GRM1*, the genes that were downregulated at all time points, have not previously been linked specifically to cardiac hypertrophy but based on our results may merit further investigation in this context.

There is also a lack of data in the literature from the early phase (8 h) of hypertrophy induction. Interestingly, we found that the highest number of DEGs was observed at the 8 h time point. We could also show that this time point had the most DEGs associated with the GO term muscle system processes.

These novel findings reveal that, on a gene level, the hypertrophic response is apparent early, which can hopefully result in a better mechanistic understanding of the initiation of hypertrophy. Possibly, this may also represent a state where therapeutic interventions may have their best opportunity to slow down or stop the progression of pathological cardiac hypertrophy. Using this model for target identification may provide novel avenues for drug discovery.

To focus our analysis, 399 genes annotated with the broader term ‘muscle system process’ including genes involved in muscle adaption, muscle contraction, muscle hypertrophy, regulation of muscle system process and relaxation of muscle were selected and analyzed in more detail. Ten percent of all genes annotated with this GO term was identified as DEGs in the ET-1 stimulated samples.

The gene expression data obtained from the RNA-seq analysis included many DEGs that are important for the development of heart diseases (Table S1). *ANKDR1* is 2.1-fold upregulated in our dataset. It is a transcription factor that is important in the myofibrillar stretch-sensor system. This marker has been found to be increased in patients with cardiomyopathy (Mikhailov and Torrado, 2008). Upregulation of this gene has been shown in stretch-induced *in vitro* models and our model replicates this finding (Herrer et al., 2014; Ovchinnikova et al., 2018). Another important gene that is observed to be upregulated in patients with heart failure is *FSTL3* (Lara-Pezzi et al., 2008). This gene is involved in cardiac remodeling, which is a hallmark of cardiac hypertrophy. Knockdown of this gene results in reduced cardiac hypertrophy in rats when exposed to hypertrophic agents (Shimano et al., 2011). In our model, the *FSTL3* gene is significantly upregulated, showing that the model can mimic the *in vivo* counterpart. One of the highly upregulated genes in our dataset was *IL-11*. It has been shown to be important for the development of fibrosis and is increased in patients with heart failure (Ye et al., 2019; Schafer et al., 2017). Inhibiting interleukin 11 could possibly be an effective treatment against several forms of heart diseases and our model could potentially be used for such investigations (Fernández-Ruiz, 2018).

The DEGs identified in our analysis after 24 h ET-1 stimulation were partly overlapping with results presented in Aggarwal et al. in which 18 h stimulation was applied (Aggarwal et al., 2014). The number of DEGs identified in each study where in the same range for both up- and downregulated genes (255 versus 235 and 244 versus 290, respectively). When investigating the overlap between these studies, we found 67 upregulated (29%) and 37 (15%) downregulated genes. In this comparison only DEGs with official gene names where considered. Of these 104 genes in total, there were two genes that were differentially expressed at all time points in our data (*COL12A1* and *DRD1*). Interestingly, *DRD1* was one of the top downregulated genes in both Aggarwal et al. and in our study. The potential role of *DRD1* in the development and progression of cardiac hypertrophy remains to be determined.

We further compared the DEGs in our dataset to a public available dataset of human myocardial biopsies with pronounced ventricular hypertrophy (Schmidt, 2010) (https://www.ebi.ac.uk/arrayexpress/experiments/E-MEXP-2296).

The DEG analysis of the *in vivo* dataset compared non-hypertrophied hearts (ejection fraction >60%) to hypertrophic hearts (ejection fraction >50%). The analysis detected nine genes that were overlapping between the *in vivo* data and our datasets (Table 1). Five of these genes codes for proteins that are known to be involved in heart disease and three of these, COL12A1, THBS1 and HSP70 are of particular interest. COL12A1 and THBS1 are genes that, when elevated, can promote fibrosis and cause cardiac remodeling, a hallmark of cardiac hypertrophy (Shirani et al., 2000; Sezaki et al., 2005). THBS1 has also been observed to be upregulated in another *in vitro* model of ET-1 induced cardiac hypertrophy (Aggarwal et al., 2014). In animal models, the heat shock protein HSP70 has been shown to be altered in the early stage of cardiac remodeling and has been suggested as an early biomarker for heart failure (Li et al., 2013). In addition, overexpression of HSP70 has been shown to induce hypertrophy in mice via activation of histone deacetylase 2 (Kee et al., 2008). Our data suggests that HSP70 also could be involved in cardiac hypertrophy in humans.

**Table 1.**
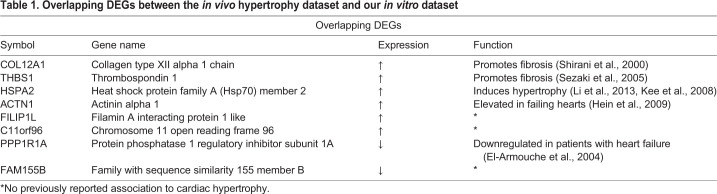
**Overlapping DEGs between the *in vivo* hypertrophy dataset and our *in vitro* dataset**

In this study, we have developed an *in vitro* model that can be used to study cardiac hypertrophy and potentially serve as a suitable platform for drug screening. We used one human cell line to generate the CMs. For future studies, it would be of interest to assess several cell lines, including cell lines derived from donors with genetic disorders associated with cardiac hypertrophy, to investigate possible differences in the hypertrophic response that may be cell line dependent. It would also be of interest to benchmark the model against human primary CMs stimulated with ET-1. However, the availability of primary human CMs is very limited, and they are difficult to maintain in culture. There is an urgent need for novel drugs targeting cardiovascular diseases, and stem cell-based models may provide new alternatives for drug discovery. Our model described here shows a robust hypertrophic response following incubation with ET-1, and it could possibly be used to screen for new drug candidates. It appears also to be suitable for dissecting the signals responsible for initiation and progression of cardiac hypertrophy in order to improve our understanding of the molecular mechanisms underlying the development of cardiac hypertrophy.

## MATERIALS AND METHODS

### Cardiomyocytes

Human CMs derived from the hiPSC line ChiPSC22 were obtained from Takara Bio (Takara Bio Europe AB). The CMs were cryopreserved at day 19 and day 32 following onset of differentiation using the STEM-CELLBANKER^®^ (cat 11890, Amsbio).

### Flow cytometry

In conjunction with the cryopreservation, 1 million cells were collected and used for flow cytometry analysis. In short, cells were fixed in paraformaldehyde (HistoLab products AB), resuspended in methanol (−20°C) (cat. 311415, Sigma-Aldrich) and stained for cardiac troponin T using Anti-cardiac Troponin T antibody (ab45932, Abcam) as primary antibody, diluted 1:500, and Alexa flour 488 Fab2 goat anti rabbit IgG antibody (ab150077, Invitrogen), diluted 1:1000, as secondary antibody. The analysis was performed using a Guava^®^ easyCyte HT Sampling Flow Cytometer (Merck Millipore).

### Hypertrophy induction

Cryopreserved CMs (from day 19 and 32 of differentiation) were thawed in CM medium [Advanced RPMI, B27 1x, Glutamax 1x, (Thermo Fisher Scientific)] supplemented with Y27632 (10 µM) (cat. Y0503, Sigma-Aldrich) and plated at 3.0×10^5^ cells/well in 48-well plate precoated with 5 µg/cm^2^ Fibronectin solution (5 µg/cm^2^) (cat. F0895, Sigma-Aldrich).

One day after thawing, the culture medium was changed to CM medium and cells were recovered for a total of 6 days before starting the experiments with ET-1. Medium (0.4 ml/cm^2^) was changed every second day. Dose response experiments were initially conducted to determine the optimum concentration of ET-1. ET-1 powder (cat. E7764, Sigma-Aldrich) was dissolved in DMSO and then added to the culture medium. The corresponding volume of DMSO was added to the control cells in parallel. For the dose-response experiment, cells were stimulated for 24 h. Subsequently, time response experiments were conducted by stimulating the CMs during different time periods. CMs that were incubated with ET-1 for more than 24 h received fresh medium with ET-1 every 24 h. All experiments were performed in triplicates and repeated three times.

### Real-time qPCR

The cells were lysed and stored in RNAprotect (cat. 76526, Qiagen) at −20°C until extraction. Total RNA was extracted using MagMAX™ Total RNA Isolation Kit (AM1830, Invitrogen, www.thermofisher.com) according to the manufacturer’s instructions and quantified by UV spectrophotometry on NanoDrop ND-1000 (NanoDrop). The quality of the RNA was verified using a 2100 Agilent Bioanalyzer. For RNA expression level analysis, RNA was converted to cDNA using High-Capacity cDNA Reverse Transcription Kit (4368814, Applied Biosystems). RT-qPCR was performed using Taqman Gene expression assays (*ACTA1* Hs00559403_m1, *NPPA* Hs00383230_g1, *NPPB* Hs00173590_m1) (Thermo Fisher Scientific) on a 7500 Fast Real-time PCR system (Applied Biosystems). Fold change values were calculated using the delta-delta CT method normalized to the CREBBP gene (Hs00173590_m1) (Holmgren et al., 2015).

### Immunocytochemistry

The cells were fixed and stained according to a previously described protocol (Ghosheh et al., 2016). Briefly, cells were fixed with paraformaldehyde (HistoLab products AB) and stained for cardiac troponin T (anti cardiac troponin T antibody, ab45932, Abcam, dilution 1:500), F-actin (Alexa Flour 488 Phalloidin, A12379, Thermo Fisher Scientific, dilution 1:500) and DNA (DAPI, cat. 62248, Thermo Fisher Scientific, dilution 1:1000). Donkey anti-rabbit Alexa flour 594 (ab150076, Abcam, dilution 1:1000) was used as secondary antibody. Images were captured with a fluorescent microscope (Eclipse TE2000-U, Nikon).

### ANP and proBNP ELISA and lactate assay

Conditioned cell culture media was collected at different time points. The media was centrifuged (5000× ***g***, 5 min) and the supernatant collected and stored at −80°C for subsequent analysis. ANP and proBNP were measured using ELISA kits (EHPRONPPB and EIAANP, Thermo Fisher Scientific) according the manufacturer’s instructions. Lactate measurements were performed with Lactate Assay kit II (cat. MAK065, Sigma-Aldrich) according to the manufacturer’s instructions. Standard curve, cell size and concentration calculations were performed using GraphPad Prism 8 (GraphPad Software Inc).

### Cell size

Cell volume measurements were performed on live cells using a Moxi Z mini automated cell counter (Orflo, www.orflo.com) with M cassettes. The medium was aspirated and the cells harvested using Trypsin/EDTA, centrifuged, resuspended in 0.5 ml DPBS (Gibco, www.ThermoFisher.com) and analyzed using the Moxi Z instrument. Cells with a diameter between 12–34 µM were included in the analysis.

### RNA-seq analysis

Library construction was performed using Illumina Truseq stranded total RNA with Illumina Ribozero method. Clustering was done by ‘cBot’ and samples were sequenced on NovaSeq6000 (NovaSeq Control Software 1.6.0/RTA v3.4.4) with a 2×51 setup using ‘NovaSeqXp’ workflow in ‘S1’ mode flowcell. The Bcl to FastQ conversion was performed using bcl2fastq_v2.19.1.403 from the CASAVA software suite. The quality scale used was Sanger/phred33/Illumina 1.8+. Processing of FASTQ files was carried out by the SciLifeLab National Genomics Infrastructure at the Uppsala Multidisciplinary Center for Advanced Computational Science, Sweden. Sequenced reads were quality controlled with the FastQC software and pre-processed with Trim Galore. Processed reads were then aligned to the reference genome of Homo sapiens (build GRCh37) with the STAR aligner. Read counts for genes were generated using the featureCounts library and normalized FPKM values calculated with StringTie. Technical documentation on the RNA-seq pipeline can be accessed here: https://github.com/SciLifeLab/NGI-RNAseq. Raw and processed data are available for download at ArrayExpress (https://www.ebi.ac.uk/arrayexpress/) accession number: E-MTAB-8548.

### Transcriptomics analysis

#### Differential expression analysis

The gene count data including 63,677 transcripts and 30 samples were imported into the R software (R Core Team, 2019) for further analysis, and statistical testing for differential expression was carried out with the quasi-likelihood F-test in the edgeR package (Robinson et al., 2009). For the DEG analysis of the *in vivo* dataset, the R package limma was used (Ritchie et al., 2015). Only genes with a 2-fold change were included in the results. A false discovery rate (FDR) rate of ≤0.05 was considered statistically significant.

To explore the overlap of DEGs between the time points, Venn diagrams of upregulated and downregulated genes respectively, were generated using InteractivVenn (Heberle et al., 2015). Heatmaps of selected sets of genes were generated using the R package pheatmap. DEGs were identified among genes annotated with the GO term GO:0003012 and the more specific GO term GO:0014898. These DEGs were used as input to Heatmapper to create heatmaps.

#### GO enrichment analysis

Gene Ontology (GO) enrichment analysis was performed using the Cytoscape software (version 3.7.1) and the ClueGO plugin (version 2.5.4). A two-sided hypergeometric test was applied with Bonferroni correction for multiple testing. GO terms at level 3–8 were included in the analysis and only terms with at least four genes with that annotation were considered. As reference set for the hypergeometric test all genes in selected ontologies were used.

### Statistical analysis

Statistical analyses of RNA (real-time qPCR), protein, lactate and cell size measurements were justified using paired sample *t*-test. A *P*-value<0.05 was considered statistically significant. The results are expressed as mean±s.d.

## Supplementary Material

Supplementary information
