# Supplementary figures and images for: Cardiac hypertrophy in a dish: a human stem cell based model
Source: Biol Open. 2020 Sep 21;9(9):bio052381. doi: 10.1242/bio.052381 (PMC7522030; doi:10.1242/bio.052381)

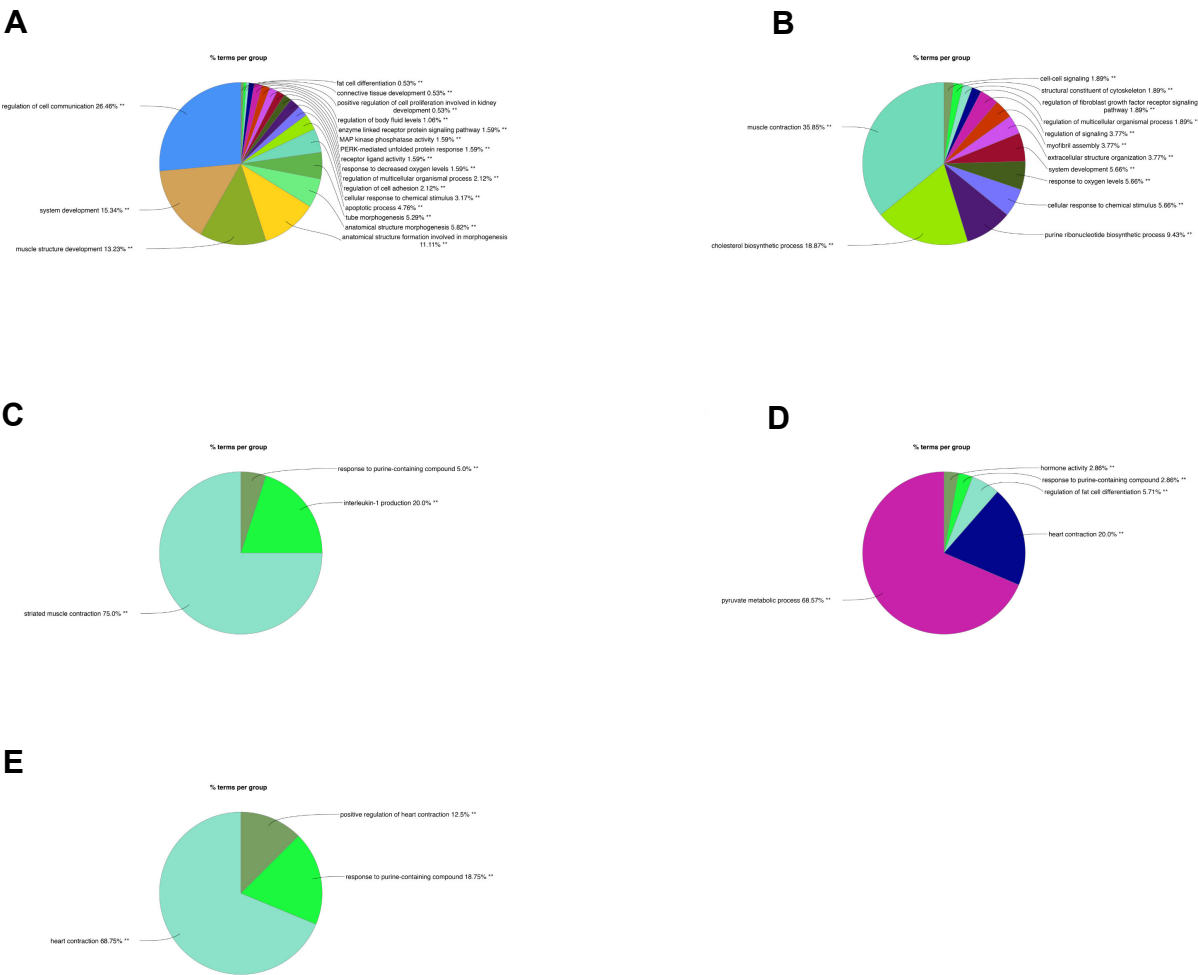

Fig S1

## Table S1

[Click here to Download Table S1](#)

Supplement: Supplementary information [file biolopen-9-052381-s1.pdf]
